# Role of Atmospheric Temperature and Seismic Activity in Spring Water Hydrogeochemistry in Urumqi, China

**DOI:** 10.3390/ijerph191912004

**Published:** 2022-09-22

**Authors:** Zhihua Zhou, Jun Zhong

**Affiliations:** China Earthquake Networks Center, Beijing 100045, China

**Keywords:** hydrogen and oxygen isotopes, hydrogeochemical, water cycle, earthquakes, springs

## Abstract

Springs offer insight into the sources and mechanisms of groundwater recharge and can be used to characterize fluid migration during earthquakes. However, few reports provide sufficient annual hydrochemical and isotopic data to compare the variation characteristics and mechanisms with both atmospheric temperature and seismic effects. In this study, we used continuous δ^2^H, δ^18^O, and major ion data from four springs over 1 year to understand the groundwater origin, recharge sources, circulation characteristics, and coupling relationships with atmospheric temperature and earthquakes. We found that (1) atmospheric temperatures above and below 0 °C can cause significant changes in ion concentrations and water circulation depth, resulting in the mixing of fresh and old water in the aquifer, but it cannot cause changes in δ^2^H and δ^18^O. (2) Earthquakes of magnitude ≥ 4.8 within a 66 km epicentral distance can alter fault zone characteristics (e.g., permeability) and aggravate water–rock reactions, resulting in significant changes in δ^2^H, δ^18^O, and hydrochemical ion concentrations. (3) Hydrogen and oxygen isotopes are the most sensitive precursory seismic indicators. The results of this study offer a reference for the establishment of long-term hydrochemical and isotopic monitoring, with the potential for use in earthquake forecasting.

## 1. Introduction

Springs offer abundant information related to deep fluids, groundwater circulation, and tectonic activity [1,2,3,4]. They may result from upwelling magmatic fluids or from deep-circulating meteoric water migrating along faults [5,6,7]. Generally, groundwater experiences continuous physical and chemical interactions during circulation; however, in relatively stable aquifers, it can maintain its specific hydrogeochemical characteristics and isotopic composition [8,9]. Earthquakes can change the crustal structure at local or regional scales, leading to the alteration of pore pressure within rock bodies and the mixing of aquifers. This process can alter the hydrogeochemistry and isotopes of spring water [10,11]. Therefore, the hydrogeochemical and isotopic characteristics of springs may reveal the origin, properties, migration path, and vertical deep circulation characteristics of the water body, including the dynamic processes of groundwater during tectonic activity [3,12,13,14,15,16,17,18,19,20,21].

Studies on spring hydrogeochemistry and isotopic composition have been reported for over 50 years. Some have focused on their changing characteristics in relation to atmospheric temperature, while others have focused on the impact of earthquakes [22,23,24,25,26,27]. However, few reports provide sufficient annual hydrogeochemical and isotopic composition data to correlate the variation characteristics with both atmospheric temperature and seismic effects. To distinguish the impacts of these two factors, it is necessary to obtain long-duration continuous observations that reveal geological structure, fluid origins, fluid circulation characteristics, interaction processes between deep and shallow fluids, and the geochemical background of the system [28,29].

In the Tianshan area with frequent earthquakes, four springs, which are located at a close surface distance, sometimes show different hydrochemical characteristics before historical earthquakes. The studies of their genetic mechanisms, influence factors, and coupled with earthquakes are quite necessary for proposing the typical indicators of earthquake forecasting and achieving disaster reduction. In this study, based on 1 year of continuous observations of hydrogeochemistry and δ^2^H–δ^18^O at four springs in Urumqi, China, we aimed to ① identify the origin and deep circulation processes of the spring water, ② reveal the influence of atmospheric temperature on groundwater circulation, and ③ establish the response relationship between geochemical changes and earthquakes. Our results offer a reference for the establishment of long-term hydrochemical and isotopic monitoring and offer new insight into precursory earthquake signals.

## 2. Geological and Hydrogeological Settings

**Figure 1:**
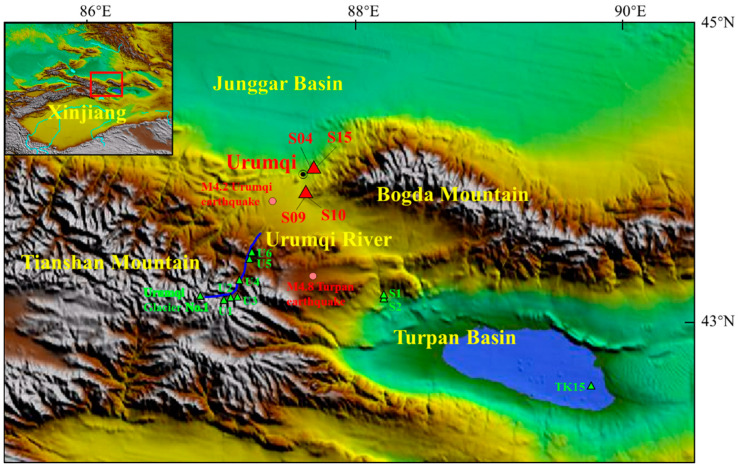
Topographical map of the study area. Red triangles show the locations of the springs analyzed in this study; green triangles show sample points from previous studies [34,35]; and pink circles show earthquakes. The inset map shows the wider regional setting with the red box delineating the study area.

Urumqi is located in the central North Tianshan Mountains of northwestern China. It sits on the southern margin of the Junggar Basin and is bounded by Bogda Mountain to the east and Turpan Basin to the southeast (Figure 1). The distance between Urumqi and the sea is the greatest of any city in the world; the city lacks groundwater resources and experiences huge annual temperature variations. The annual average temperature is 7.88 °C, while the monthly average temperatures in summer (July to August) and winter (December to January) are 24 °C and −26.5 °C, respectively. This extreme temperature difference can reach 69.2 °C [30].

The research area covered four springs (Spring 04, Spring 09, Spring 10, and Spring 15) located in the Permian fan delta shore shallow lake facies on the edge of Bogda Mountain [31]. Spring 04 and 10 always have bubbles before earthquakes, which indicates that they are closely related to the fault. The topography of this area is characterized by a piedmont plain with high elevations to the south and low elevations to the north. Groundwater flows from south to north, and mainly originates from Urumqi Glacier No. 1 in the southwest of the study area [32]. However, according to the local hydrogeological conditions of Spring 09 and Spring 10, groundwater also flows from mountains in the east towards the piedmont plain in the west. Groundwater is mostly stored in bedrock fissures, which is easily controlled by vertical climate zoning and geological structures [33].

Spring 04 (87.66° E, 43.83° N) and Spring 15 (87.66° E, 43.83° N) are ~0.8 km altitude and located 300 m apart on the Shuimogou–Baiyangnangou fault. The lithology of the Spring 04 aquifer is Permian oil shale and siliceous sandstone, and the hydrochemical type is SO_4_–Na. The average annual water temperature is ~20 °C, and the pH is 9.29. The aquifer of Spring 15 is Permian sandstone and thin limestone containing fissure phreatic water; the hydrochemical type is SO_4_·Cl–Na·Ca or SO_4_–Na·Ca. The annual average water temperature is ~10 °C, the flow is 1.5 L/s, and the pH is 7.6.

Spring 09 (87.62° E, 43.70° N) and Spring 10 (87.62° E, 43.70° N) are ~1 km altitude and located 100 m apart in the Northwest Liushugou–Hongyanchi fault zone (Figure 1), which mainly runs through Carboniferous and Permian strata with strong folds in the south, thrusting northward onto Permian and Triassic strata. The lithology of the Spring 09 aquifer is Permian sandstone and conglomerate; the bedrock fissure water has a hydrochemical type of SO_4_·Cl–Na or SO_4_·HCO_3_–Na. The average annual water temperature is ~10.6 °C, and the pH is 8.0. The aquifer of Spring 10 is Permian siliceous sandstone and conglomerate along the crushed zone of the fault. The hydrochemical type is SO_4_·Cl–Na, the average annual water temperature is ~11.2 °C, and the pH is 7.7.

## 3. Samples and Methods

We collected 200 samples from four springs (springs 04, 15, 09, and 10) over the course of ~1 year. Samples were collected every 2 days during three sampling periods: 15 March to 30 April 2020, 3 September to 3 October 2020, and 15 December 2020 to 11 January 2021. Atmospheric temperature was > 0 °C (T > 0 °C) from 15 March to 3 October 2020 and <0 °C (T < 0 °C) from 15 December 2020 to 11 January 2021.Water samples were stored in 100 and 30 mL polyethylene bottles for major ion (Na^+^, K^+^, Ca^2+^, Mg^2+^, Cl^−^, SO_4_^2−^, NO_3_^−^, HCO_3_^−^, CO_3_^2−^, F^−^) and isotope (δ^2^H and δ^18^O) analyses, respectively. All samples were measured at the Institute of Surface-Earth System Science, Tianjin University.

The δ^2^H and δ^18^O were analyzed using a liquid water isotope analyzer (Picarro L2140-I, Santa Clara, CA, USA) after filtration through a 0.22 μm cellulose-acetate filter membrane [36]. Based on replicate measurements of standards and samples, the analytical precisions for δ^2^H and δ^18^O were better than ±0.46‰ and ±0.05‰, respectively. The results are reported relative to the Vienna Standard Mean Ocean Water (V-SMOW). Extremely accurate measurements of isotopic ratios were achieved when three standard samples were measured after every seven unknown samples. 

Samples for major ion analysis were filtered through a 0.45 μm Millipore membrane. Major cations (Na^+^, K^+^, Ca^2+^, and Mg^2+^) were measured by an inductively coupled plasma emission spectrometer and major anions (Cl^−^, SO_4_^2−^, F^−^, and NO_3_^−^) were measured by ion chromatography (Thermo Aquion) with analytical error of < 1 mg/L [37]. HCO_3_^−^ and CO_3_^2−^ concentrations were measured using standard titration procedures with a ZDJ-100 potentiometric titrator (reproducibility within ± 2%). The normalized inorganic charge balance varied within ± 5%, indicating the accuracy of the measured data. 

## 4. Results

### 4.1. Hydrochemical Characteristics

**Figure 2:**
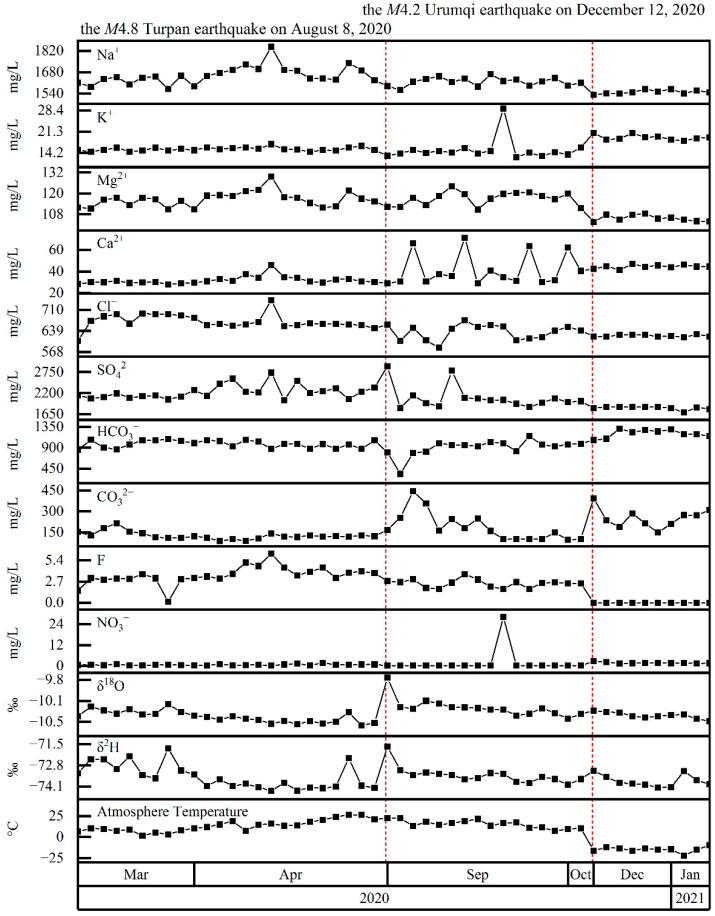
Time series of Spring 04 major ion concentrations (Na^+^, K^+^, Mg^2+^, Ca^2+^, Cl^−^, SO_4_^2−^, HCO_3_^−^, CO_3_^2−^, F^−^, and NO_3_^−^), stable isotopes (δ^2^H, δ^18^O), and atmospheric temperature. Red-dashed lines mark the occurrence of potentially significant earthquakes within the study area.

Water samples from Spring 04 were very high in Na^+^ (1531–1848 mg/L) and SO_4_^2−^ (1698–2899 mg/L); the hydrochemical type was SO_4_–Na (Figure 2). HCO_3_^−^ increased significantly in winter and reached 1065–1308 mg/L; however, the hydrochemical type did not change significantly, with only a small number of samples having a SO_4_·HCO_3_–Na hydrochemical type. The δ^2^H and δ^18^O gradually decreased over time, interrupted by stepwise increases coincident with the M4.8 Turpan earthquake on 8 August 2020, after which values returned to a declining trend. However, stable isotopes showed no relationship with atmospheric temperature (i.e., above or below 0 °C) or the M4.2 Urumqi earthquake on 12 December 2020.

**Figure 3:**
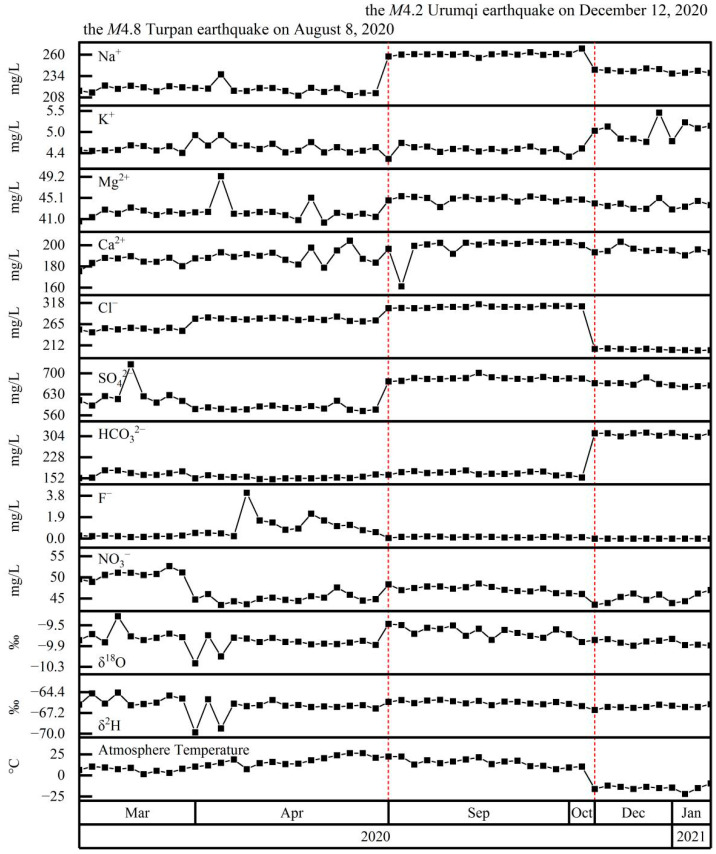
Time series of Spring 15 major ion concentrations (Na^+^, K^+^, Mg^2+^, Ca^2+^, Cl^−^, SO_4_^2−^, HCO_3_^−^, F^−^, and NO_3_^−^), stable isotopes (δ^2^H, δ^18^O), and atmospheric temperature. Red-dashed lines mark the occurrence of potentially significant earthquakes within the study area.

Spring 15 samples contained Na^+^ of 210–268 mg/L, Ca^2+^ of 161–205 mg/L, SO_4_^2−^ of 574–730 mg/L, and Cl^−^ of 199–315 mg/L (Figure 3). With winter atmospheric temperature, HCO_3_^−^ (301–317 mg/L) increased and Cl^−^ decreased. As a result, the hydrochemical type was SO_4_·Cl-Na·Ca when the atmospheric temperature was >0 °C, and SO_4_–Na·Ca when atmospheric temperature was <0 °C. Changes in δ^2^H and δ^18^O were similar to those of Spring 04; that is, they decreased over time, interrupted by stepwise increases coincident with the M4.8 earthquake. Again, the stable isotopes showed no clear relationship with atmospheric temperature (i.e., above or below 0 °C) or the M4.2 Urumqi earthquake on 12 December 2020.

**Figure 4:**
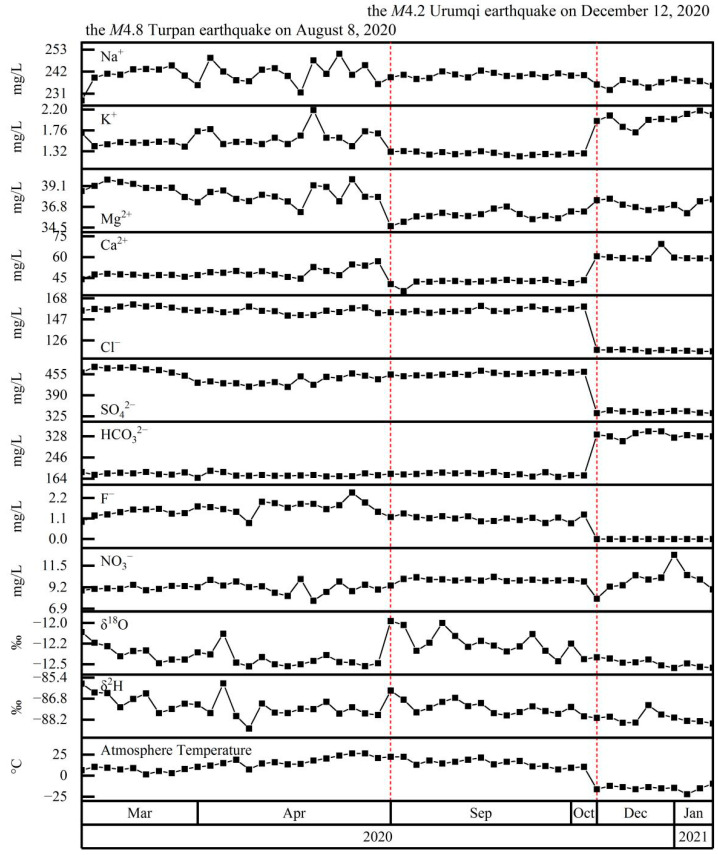
Time series of Spring 09 major ion concentrations (Na^+^, K^+^, Mg^2+^, Ca^2+^, Cl^−^, SO_4_^2−^, HCO_3_^−^, F^−^, and NO_3_^−^), stable isotopes (δ^2^H, δ^18^O), and atmospheric temperature. Red-dashed lines mark the occurrence of potentially significant earthquakes within the study area.

The main hydrochemical ions of Spring 09 were Na^+^ (210–423 mg/L), SO_4_^2+^ (334–730 mg/L), and Cl^−^ (115–425 mg/L) (Figure 4). For samples collected at T < 0 °C, the concentrations of HCO_3_^−^ (309–348 mg/L) and Ca^2+^ (59–70 mg/L) increased, while Cl^−^ and SO_4_^2−^ decreased. As a result, when the atmospheric temperature was >0 °C, the hydrochemical type was SO_4_·Cl–Na, but when the atmospheric temperature was <0 °C, the hydrochemical type was SO_4_·HCO_3_–Na. As with the other springs, δ^2^H and δ^18^O decreased over time, interrupted by stepwise increases coincident with the M4.8 earthquake. The stable isotopes showed no clear relationship with atmospheric temperature (i.e., above or below 0 °C) or the M4.2 Urumqi earthquake on 12 December 2020.

**Figure 5:**
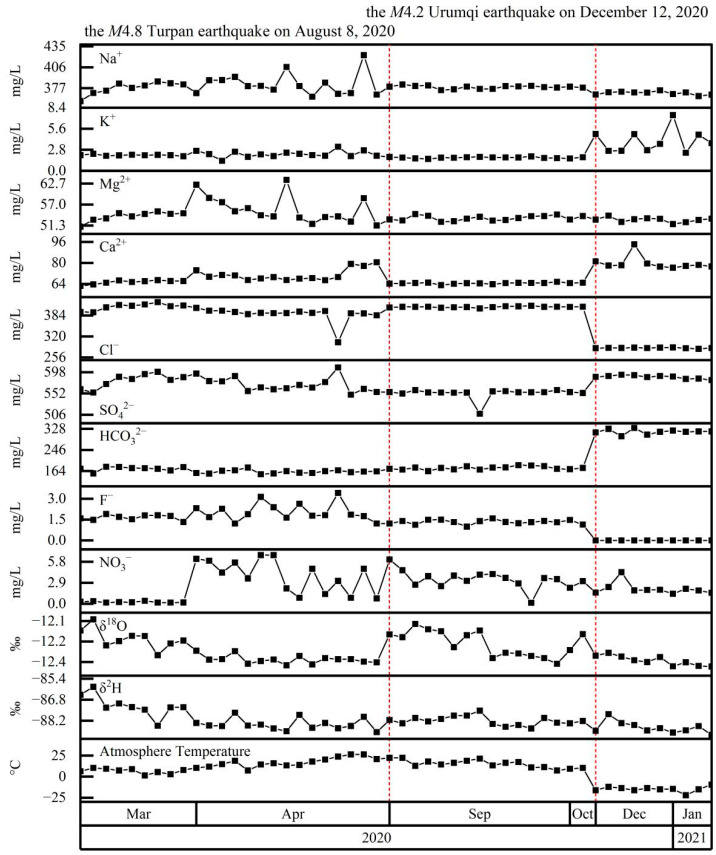
Time series of Spring 10 major ion concentrations (Na^+^, K^+^, Mg^2+^, Ca^2+^, Cl^−^, SO_4_^2−^, HCO_3_^−^, F^−^, and NO_3_^−^), stable isotopes (δ^2^H, δ^18^O), and atmospheric temperature. Red-dashed lines mark the occurrence of potentially significant earthquakes within the study area.

Spring 10 was similar to Spring 09; the main ions were Na^+^ (210–423 mg/L), SO_4_^2−^ (508–730 mg/L), and Cl^−^ (199–425 mg/L), and when atmospheric temperature was >0 °C, the hydrochemical type was SO_4_·Cl–Na or Cl·SO_4_–Na (Figure 5). However, when the atmospheric temperature was <0 °C, the concentrations of HCO_3_^−^ (299–332 mg/L), Ca^2+^ (77–95 mg/L), and K^+^ (2–5 mg/L) increased, while Cl^−^ decreased. Therefore, when the atmospheric temperature was <0 °C, the hydrochemical type was SO_4_·Cl-Na. As with the other springs, δ^2^H and δ^18^O decreased over time, interrupted by stepwise increases coincident with the M4.8 earthquake. The stable isotopes showed no clear relationship with atmospheric temperature (i.e., above or below 0 °C) or the M4.2 Urumqi earthquake on 12 December 2020.

In summary, although the major ion concentrations of the four springs differed, all had elevated Na^+^, SO_4_^2−^, and Cl^−^. Moreover, when the atmospheric temperature fell below 0 °C in winter, the concentrations of HCO_3_^−^, Ca^2+^, and K^+^ increased, while Cl^−^ and Na^+^ decreased; changes in summer were not synchronous. In contrast, isotopes were not affected by atmospheric temperature but did show obvious stepwise increases associated with the M4.8 Turpan earthquake in 2020.

**Figure 6:**
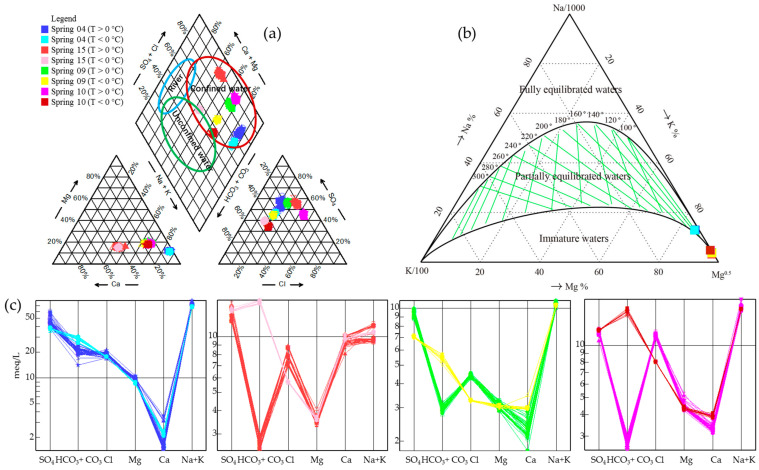
Hydrogeochemistry of spring water samples. (**a**) Piper diagram, with water types within the oval ranges from previous studies [35]. (**b**) Na–K–Mg ternary diagram and (**c**) Schoeller diagram showing the major ion characteristics of each spring.

Piper diagram analysis confirmed the changes in water hydrogeochemistry between winter and summer (Figure 6, Table 1). It also showed that all the samples from springs 04 and 09, and most from springs 10 and 15, were water from a confined aquifer. However, when the atmospheric temperature was <0 °C, samples from springs 10 and 15 fell within the boundary zone of confined and unconfined aquifers (Figure 6a).

Based on the Na–K–Mg ternary diagram (Figure 6b), water from Spring 04 was classified as deep geothermal water partially equilibrated with the host rock; samples from the other springs were classified as shallow geothermal water non-equilibrated with the host rock. A Schoeller diagram (Figure 6c) showed no hydraulic connection between the four springs, but also confirmed different aquifer characteristics depending on atmospheric temperature (above or below 0 °C).

### 4.2. Geothermometry and Circulation Depth

We applied the Na–K–Ca geothermometer according to the following empirical formula [38]:

(1) $$T_{\mathrm{Na}\text{-}K\text{-}\mathrm{Ca}}=\frac{1647}{\mathrm{lg}\left(\mathrm{Na}/K\right)+0.25\;\times \;\beta \left[\mathrm{lg}(\sqrt{\mathrm{Ca}}/\mathrm{Na}+2.06)\right]+2.47}-273.15$$

where *β* = 0.75 (*T* < 100 °C) and *β* = 0.25 (*T* > 100°C).

The groundwater circulation depth was calculated as [39]:

(2) $${H=(T}_{z}-T_{0}{)/G+H}_{0}$$

where *H* is the circulation depth (m), *T*_Z_ the estimated reservoir equilibrium temperature (°C), *T*_0_ is the local annual temperature (°C), *G* is the thermal gradient (°C/km), and *H*_0_ is the thickness of the constant temperature zone (m). The constant temperature zone is defined as the subsurface depth at which changes in atmospheric temperature have no effect on the temperature of the zone [40]. From previous studies, we chose values of *H*_0_ = 20 m, *T*_0_ = 18 °C, and *G* = 18.2 °C/km [39,41]. Circulation depths were estimated for both summer and winter (atmospheric temperatures of > and < 0 °C, respectively; Table 1).

Differences in reservoir temperature estimates reached 2.4–9 °C between winter and summer (Table 1), reflecting significant seasonal differences in circulation depth. The circulation depth of Spring 04 was the deepest (4710–5210 m); those of Spring 09, Spring 10, and Spring 15 were within 880–1940 m (Table 1).

### 4.3. δ2H and δ^18^O Characteristics

**Figure 7:**
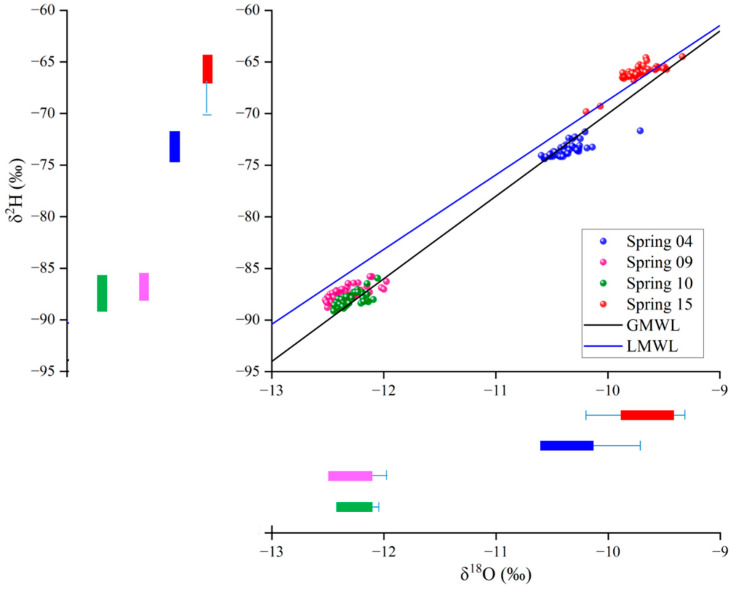
Bivariant δ^2^H–δ^18^O plot for samples from Spring 04 (blue circles), Spring 15 (red circles), Spring 09 (pink circles), and Spring 10 (green circles). The global meteoric water line (GMWL of Craig [42]) is shown by the black line and indicates meteoric water affected by water–rock interaction. The local meteoric water line (LMWL of Feng et al. [43]) is shown by the blue line.

The δ^2^H and δ^18^O values of each individual spring were relatively concentrated (Figure 7) and had no significant relationship with atmospheric temperature of > or <0 °C. Samples from Spring 15 plotted on the Xinjiang local meteoric water line (LMWL, δ^2^H = 7.23δ^18^O + 3.60); samples of springs 04, 09, and 10 fell below the LMWL, possibly indicating water–rock isotope exchange resulting in “oxygen drift”. 

Hydrogen and oxygen isotopes in atmospheric precipitation are affected by altitude; therefore, isotopes can be used to estimate the altitude of meteoric water involved in groundwater recharge. In western China, recharge altitude can be calculated as:

δ^2^H = −0.026*H* − 30.2 (3)

where *H* is the recharge altitude of the springs (m). The results show that the average recharge altitudes of the four springs were not significantly different in summer and winter (Table 1).

### 4.4. Coupling between Hydrochemistry and Earthquakes

The Dobrovolsky et al. [44] formula was used to identify earthquakes with the potential to cause precursory signals at the four springs:

R = 10^0.43 M^ (4)

where R is the radius of the effective precursory manifestation area depending on earthquake magnitude. We identified two earthquakes—the Turpan M4.8 earthquake (87.67° E, 43.23° N) on 8 August 2020 and Urumqi M4.2 earthquake (87.37° E, 43.65° N) on 12 December 2020—with the potential to affect continuous hydrochemical observations at the four springs (http://data.earthquake.cn; Figure 1). The Turpan M4.8 earthquake was caused by strike-slip fault motion within the eastern segment of the Tianshan earthquake zone; the epicenter was ~52–66 km from the springs. The Urumqi M4.2 earthquake was caused by reverse fault motion within the central section of the Tianshan earthquake zone; the epicenter was ~20–31 km from the springs. Of the two earthquakes, only one, the M4.8 Turpan earthquake of 8 August 2020, had a clear temporal correlation with sudden changes in isotope signatures, suggesting a coupling between hydrochemical changes and these earthquakes. 

## 5. Discussion

### 5.1. Groundwater Origin, Recharge Sources, and Circulation Characteristics

Hydrogen and oxygen isotopes are affected by meteorological processes; as such, the values and distribution characteristics of δ^2^H and δ^18^O provide a basis for the investigation of groundwater recharge sources [4]. Samples from the four springs are all consistent with either the GMWL or LMWL, indicating the strong influence of meteoric water (Figure 7).

Springs 04 and 15 are only 300 m apart within the same structural setting. The samples show some similarities; for example, both have higher δ^2^H and δ^18^O values than those from springs 09 and 10, reflecting greater rainfall recharge [45]. However, Spring 04 samples plot on the GMWL, to the right of the LMWL, indicating water–rock interaction. In contrast, Spring 15 plots to the left of the LMWL, which can be explained by degassing or by a large deuterium excess related to the climate regime at the time of precipitation [26]. Moreover, the data for Spring 15 suggest relatively shallower groundwater circulation and with more rapid circulation speeds than that of Spring 04 (Figure 7). As with Spring 04, Springs 09 and 10 plot on the GMWL, to the right of the LMWL, indicating water–rock interaction. Compared with Spring 09, positive shifts in δ^18^O for Spring 10 reflect a strong water–rock interaction related to high temperature. 

In addition, the average altitude of recharge water is much higher than the actual altitude, which should be long-distance runoff recharge (Table 1). Therefore, the water at all four springs is mainly supplied by atmospheric precipitation and snowmelt from surrounding mountains [26,46]. However, during circulation, this water undergoes water–rock interactions, during which it takes on soluble ions from the aquifer rocks [34]. Differences in the geochemical and structural conditions of the aquifers (e.g., lithology, weathering, structural fissures) lead to differences in the leaching, erosion, and infiltration processes, resulting in variation among the springs. 

**Figure 8:**
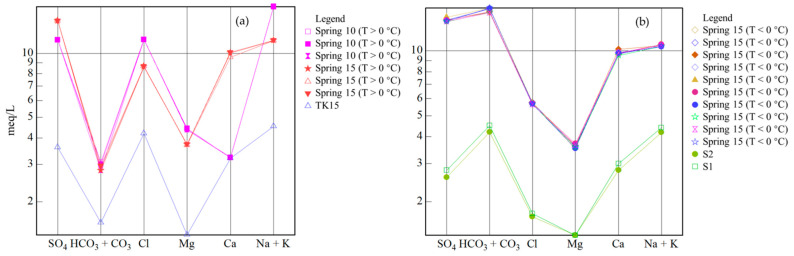
Schoeller diagrams of samples from Spring 15 and springs in the Turpan Basin [35]. (**a**) Spring 15 samples collected at T > 0 °C and the TK15 sample from Turpan Basin; (**b**) Spring 15 samples collected at T < 0 °C and samples from springs S1 and S2 in the Turpan Basin.

Groundwater in the Urumqi region flows from south to north, and mainly originates from Urumqi Glacier No.1 in the southwest [32]. River and precipitation samples from the region (i.e., samples with no hydraulic connection to the springs in this study) have high Ca^2+^ and HCO_3_^−^ concentrations, with both being the major ions in the hydrochemical types [47]. In general, all four springs are fed by confined aquifers (Figure 6). However, some samples of Spring 15 (T > 0 °C) have a hydraulic connection with unconfined aquifer sample TK15 (89.77° E, 42.62° N) from the Turpan Basin, while others (T < 0 °C) are hydraulically related to springs S1 (88.21° E, 43.12° N) and S2 (88.21° E, 43.11° N) in the Turpan Basin [35] (Figure 8). This suggests long-distance runoff recharge from the Turpan Basin, which is also consistent with the high Cl^−^ concentrations, which result from rock salt dissolution and long runoff. In addition, groundwater flow also occurs from mountains in the east towards the piedmont plain in the west, as evidenced by the local hydrogeological conditions of Spring 09 and Spring 10. The water origin of the Turpan Basin is directly related to Bogda Mountain. Furthermore, the average altitude of Bogda Mountain is ~4 km, which is consistent with the calculated result in Table 1. In summary, the origin of water in the four springs is most likely rainfall and the deep circulation of meteoric water from Bogda Mountain in the east.

**Figure 9:**
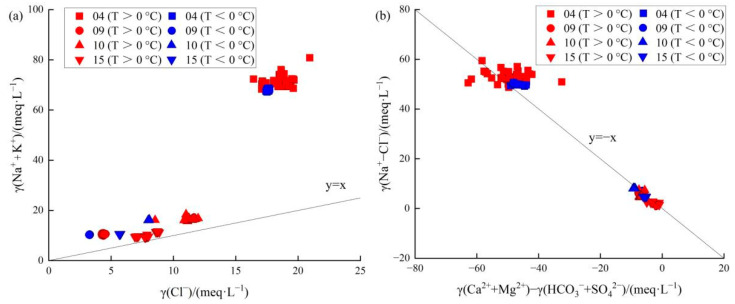
Ion concentration relationships for samples from the four springs. (**a**) γ(Na^+^ + K^+^)/γ(Cl^−^) and (**b**) γ(Na^+^ − Cl^−^)/[γ(Ca^2+^ + Mg^2+^) − γ(HCO_3_^−^+SO_4_^2−^)].

Regardless of atmospheric temperature (> or <0 °C), γ(Na^+^ + K^+^)/γ(Cl^−^) was >1 for all samples from all four springs, indicating that Na+ in the water comes from weathering dissolution or cation exchange of silicate minerals (Figure 9a). In terms of γ(Na^+^ − Cl^−^)/[γ(Ca^2+^ + Mg^2+^)-γ(HCO_3_^−^ + SO_4_^2−^)], most samples from Spring 04 were above the y = −x line, indicating enhanced cation exchange, especially at T < 0 °C with increased circulation depth (Figure 9b). Spring 09 samples for T > 0 °C fell below the y = −x line, indicating that they are less affected by cation exchange, while samples for T > 0 °C fell on the y = −x line, indicating that cation exchange is significant. In contrast, almost all samples from springs 10 and 15 fell below the y = −x line, indicating that they are less affected by cation exchange and more impacted by silicate dissolution, regardless of atmospheric temperature (i.e., > or <0 °C). The altitude of spring rainfall was not season-dependent, but circulation depth was (Table 1). Both Spring 04 and Spring 09 had increased circulation depth and increased cation exchange during winter. However, the circulation depths of springs 10 and 15 increased without significant cation exchange, and the water source was still the original aquifer.

The high Na^+^, Cl^−^, and SO_4_^2−^ concentrations of all four springs also reflect the long runoff and deep circulation characteristics. Under the action of gravity, circulation depths reached ~0.88–5.21 km (Table 1). After being heated by high-temperature rocks, the water mixed with fluids from the deep crust and then moved upwards along faults and fractures. Spring 04 and Spring 15 are 300 m apart on the surface, but exhibit significant differences in circulation depth. The circulation path of Spring 04 is more closely related to the fault zone, resulting in deeper circulation. The surface distance between Spring 09 and Spring 10 is just 100 m, but the depths of deep circulation, especially for samples collected at T > and < 0 °C, differ, reflecting differences in fractures, porosity, and permeability. In summary, Spring 04 and Spring 10 are more influenced by deep circulation compared with Spring 09 and Spring 15. 

The Schoeller diagram shows that the four springs have different recharge sources at T < 0 °C and T > 0 °C (Figure 6), indicating the complexity of the geological structure in the study area. In this region, sedimentary strata are underlain by granite [48]; during deep winter circulation, this granite releases Na^+^, Ca^2+^, and HCO_3_^−^ ions. However, temporal variations in hydrogen and oxygen isotopes show that the water recharge source did not change significantly. The δ^2^H–δ^18^O plots of springs 04, 09, and 10 are all located on the right side of the LMWL (Figure 7), indicating the mixing of fresh and old water in the aquifer [46]. Water–rock interaction occurs by the precipitation and/or dissolution of minerals [49]; provided this occurs stoichiometrically, species ratios are fixed by the stoichiometry of the ongoing precipitation/dissolution reactions [26]. As such, ion concentration ratios can distinguish groundwater sources from water–rock interactions. However, if ion concentration ratios represent nonstoichiometric precipitation and/or dissolution of minerals, it suggests that atmospheric temperature caused mixing rather than water–rock reactions.

In summary, the groundwater origin of the four springs is mainly geothermally-heated, deep circulated atmospheric precipitation, and snowmelt; however, there is also a contribution from long-distance basin recharge sources, which cause increases in dissolved solids. Water–rock reactions are dominated by the dissolution of silicate minerals. Seasonal atmospheric temperature changes have a great impact on the circulation depth of the four springs, and the δ^2^H–δ^18^O data show that changes in ion concentrations are the result of mixing rather than water–rock reactions. Meanwhile, samples collected at T < 0 °C reflect the mixing of fresh and old water in the aquifer.

### 5.2. Hydrochemical Changes Coupled to Earthquakes

The M4.8 Turpan earthquake (8 August 2020), which occurred during a period of stable atmospheric temperature, had a significant impact on isotope signals and ion concentrations. In contrast, the M4.2 Urumqi earthquake (12 December 2020) occurred at the same time as a large change in atmospheric temperature (from ~10 to −16 °C) and was related to a stepwise change in ion concentrations but little change in the isotope signal.

**Figure 10:**
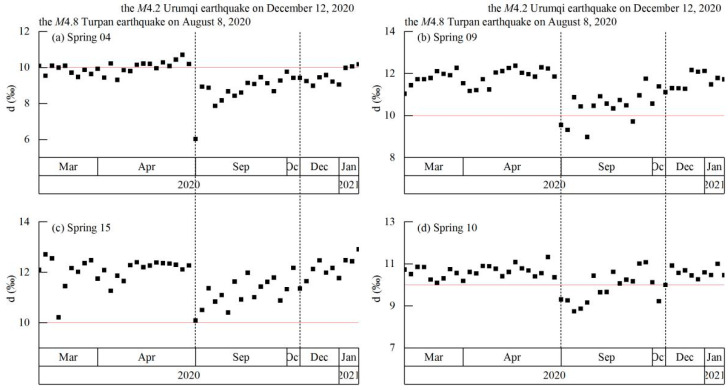
Time series of δ^2^H–δ^18^O deuterium excess (where d = δ^2^H – 8 × δ^18^O) of spring samples. The red line at ordinate = 10 shows the δ^2^H–δ^18^O deuterium excess of the global meteoric water line (GMWL). Black dashed lines denote earthquakes.

In general, groundwater moves slowly through the aquifer system under hydrological and geological processes; as such, hydrogeochemical changes tend to be gradual. However, as seismic activity can cause sudden changes to aquifers and the surrounding rock (e.g., changes in permeability or water mixing), the resulting hydrogeochemical changes can occur rapidly. We calculated the time series of the δ^2^H–δ^18^O deuterium excess (where d = δ^2^H − 8×δ^18^O) and compared it with that of the GMWL (Figure 10). The δ^2^H–δ^18^O deviated significantly at the time of the M4.8 Turpan earthquake, reflecting a change in water source coupled with the occurrence of an earthquake. In contrast, δ^2^H–δ^18^O did not change significantly at the time of the M4.2 Urumqi earthquake; that is, the water source continued to be controlled by meteoric water. The changes in ion concentrations reflected the change in atmospheric temperature, and were not related to the earthquake. 

A seismic observation well (X10; 87.62° E, 43.70° N) near Spring 10 experienced a coseismic step change of water level during the M4.8 Turpan earthquake (52 km from the epicenter) [33]. This confirms that coseismic static strains in this region were sufficiently strong to alter pore fluid pressures and permeability. In contrast, the M4.2 Urumqi earthquake, with an epicenter just 13 km from X10, did not cause a coseismic step change in the water level of the well. This may indicate that the energy of the M4.2 earthquake was insufficient to cause a change in permeability. These findings support our conclusions; that is, the M4.8 Turpan earthquake affected both ion concentrations and δ^2^H–δ^18^O, but the M4.2 Urumqi earthquake did not; changes coincident with the second event were related to changes in atmospheric temperature.

According to the geological and hydrogeological setting, the four observation springs are located in areas where stress is easy to concentrate, making them sensitive to seismic activity. The ion concentrations increased slowly before the M4.8 Turpan earthquake, possibly owing to large-scale loading of regional stress and changes in fractures within the fault zone. This allowed a high concentration of fluid to enter the springs and change the ion concentrations; moreover, this increase in permeability would also have intensified water–rock reactions within fractures. In general, owing to the pumping effect of earthquakes, shallow groundwater can also diffuse to the deeper fault, and the circulation depth of groundwater can change before and after earthquakes [50]. However, the M4.8 Turpan earthquake did not cause such changes in the springs, and mixing with shallow water can be ignored. Changes in Cl^−^ concentration related to earthquakes can reflect changes in runoff fractures. The coupling relationship between Cl^−^ concentration and the M4.8 Turpan earthquake is consistent with the δ^2^H–δ^18^O response. This suggests a high concentration of fluid input and a strong possibility of mixing with old water. On the whole, ion concentrations in the springs increased before the earthquake while the δ^2^H–δ^18^O data drifted towards the right of the plot, indicating that water–rock reactions intensified, solubility increased, and new fracture surfaces appeared in the aquifer or fault zone.

## 6. Conclusions

In this study, the hydrogeochemistry (major ion concentrations and δ^2^H and δ^18^O isotopes) of four springs in the Urumqi area was analyzed for a 1-year period. We conclude that the four springs are likely recharged by deep circulation of meteoric water from Bogda Mountain in the east, as well as long-distance runoff recharge from the Turpan Basin to the south. The hydrochemical type and circulation depth of the springs are both affected by atmospheric temperature (i.e., T> and <0 °C), although the source remains the same (i.e., meteoric water).

We conclude that seasonal changes in atmospheric temperature and M ≥ 4.8 earthquakes within 66 km can cause changes in the spring water ion concentrations, but only earthquakes can cause changes in stable isotopes; this suggests that mixing rather than water–rock reactions is coupled with atmospheric temperature (i.e., T> and <0 °C). Ion concentrations and δ^2^H–δ^18^O are sensitive to earthquakes of M ≥ 4.8, which can alter fault zone characteristics (e.g., permeability and fractures) and intensify water–rock reactions. 

The results suggest that continuous spring hydrogeochemical observations, especially stable isotopes, offer potential precursory information before earthquakes. Moreover, such an approach offers the potential for a better understanding of the coupling between seismic activity and geochemical variations.

## Figures and Tables

**Table 1:** Estimated aquifer characteristics.

	Hydrochemical Type	Average Altitude of Recharge Water (km)	Temperature (Na-K-Ca Geothermometer; °C)	Average Circulation Depth (km)
Spring 04 (T > 0 °C)	SO_4_–Na	3.99	103.4	4.71
Spring 04 (T < 0 °C)	SO_4_–Na SO_4_·HCO_3_–Na	4.00	112.4	5.21
Spring 15 (T > 0 °C)	SO_4_·Cl–Na·Ca SO_4_·Cl–Ca·Na	3.70	38.04	1.12
Spring 15 (T < 0 °C)	SO_4_–Na·Ca	3.71	40.43	1.25
Spring 09 (T > 0 °C)	SO_4_·Cl–Na	4.52	33.67	0.88
Spring 09 (T < 0 °C)	SO_4_·HCO_3_–Na	4.55	36.83	1.05
Spring 10 (T > 0 °C)	SO_4_·Cl–Na Cl·SO_4_–Na	4.55	39.06	1.18
Spring 10 (T < 0 °C)	SO_4_·Cl–Na	4.57	52.93	1.94

## Data Availability

All data are available from the corresponding author upon reasonable request.

## References

[1] Chen L.W., Yin X.X., Liu X. (2013). Tracing of recharge sources of deep aquifers in the concealed type colliery of North China by hydrochemistry and isotopes. Sci. Geogr. Sinica.

[2] Clark I.D., Fritz P. (1997). Environmental Isotopes in Hydrogeology.

[3] Huang P.H., Chen J.S. (2012). Recharge sources and hydrogeochemical evolution of groundwater in the coal-mining district of Jiaozuo, China. Hydrogeol. J..

[4] Huang X., Wang G., Liang X., Cui K., Ma L., Xu Q. (2018). Hydrochemical and Stable Isotope (δD and δ^18^O) Characteristics of Groundwater and Hydrogeochemical Processes in the Ningtiaota Coalfield, Northwest China. Mine Water Environ..

[5] Kendall C., Kendall C., McDonnell J.J. (1998). Tracing nitrogen source and cycling in catchments. Isotope Tracers in Catchment Hydrology.

[6] Cook P., Herczeg A.L. (2000). Environmental Tracers in Subsurface Hydrology.

[7] Hosono T., Yamanaka C. (2021). Origins and pathways of deeply derived carbon and fluids observed in hot spring waters from non-active volcanic fields, western Kumamoto, Japan. Earth Planets Space.

[8] Kendall C., McDonnell J.J. (1998). Isotope Tracers in Catchment Hydrology.

[9] Wang P., Yu J.J., Zhang Y.C., Liu C.M. (2013). Groundwater recharge and hydrogeochemical evolution in the Ejina basin, northwest China. J. Hydrol..

[10] Song S., Ku W.Y., Chen Y.L., Liu C.M., Chen H.F., Chan P.S., Chen Y.G., Yang T.F., Chen C.H., Liu T.K. (2006). Hydrogeochemical anomalies in the springs of the Chiayi area in west-central Taiwan as possible precursors to earthquakes. Pure Appl. Geophys..

[11] Ingebritsen S., Manga M. (2014). Earthquakes: Hydrogeochemical precursors. Nat. Geosci..

[12] André L., Franceschi M., Pouchan P., Atteia O. (2005). Using geochemical data and modelling to enhance the understanding of groundwater flow in a regional deep aquifer, Aquitaine Basin, southwest of France. J. Hydrol..

[13] Gastmans D., Chang H.K., Hutcheon I. (2010). Groundwater geochemical evolution in the northern portion of the Guarani Aquifer System (Brazil) and its relationship to diagenetic features. Appl. Geochem..

[14] Moral F., Cruz-Sanjulian J.J., Olias M. (2008). Geochemical evolution of groundwater in the carbonate aquifers of Sierra de Segura (Betic Cordillera, southern Spain). J. Hydrol..

[15] Sun F.Q., Hou G.C., Dou Y., Fang C.S., Jiang J., Zhang L.Z. (2009). Hydrogeochemistry evidence of groundwater circulation features in Ordos Cretaceous basin—A case study in Chabu well field. J. Jilin U Earth Sci..

[16] Wang Y.X., Guo Q.H., Su C.L., Ma T. (2006). Strontium isotope characterization and major ion geochemistry of karst water flow, Shentou, northern China. J. Hydrol..

[17] Chiodini G., Frondini F., Cardellini C., Parello F., Peruzzi L. (2000). Rate of diffuse carbon dioxide Earth degassing estimated from carbon balance of regional aquifers: The case of central Apennine, Italy. J. Geophys. Res. Solid Earth.

[18] Yamada M., Ohsawa S., Kazahaya K., Yasuhara M., Takahashi H., Amita K., Mawatari H., Yoshikawa S. (2011). Mixing of magmatic CO_2_ into volcano groundwater flow at Aso volcano assessed combining carbon and water stable isotopes. J. Geochem. Explor..

[19] Rive K., Gaillardet J., Agrinier P., Rad S. (2013). Carbon isotopes in the rivers from the Lesser Antilles: Origin of the carbonic acid consumed by weathering reactions in the Lesser Antilles. Earth Surf. Proc. Land.

[20] Han G., Yang K., Zeng J., Zhao Y. (2021). Dissolved iron and isotopic geochemical characteristics in a typical tropical river across the floodplain: The potential environmental implication. Environ. Res..

[21] Xie T., Ye Q., Lu J. (2020). Electrical resistivity of three phase cracked rock soil medium and its anisotropic changes caused by crack changes. Geomat. Nat. Hazards Risk.

[22] Zhang W., Kang S., Shen Y., He J., Chen A. (2017). Response of snow hydrological processes to a changing climate during 1961 to 2016 in the headwater of Irtysh River Basin, Chinese Altai Mountains. J. Mt. Sci.

[23] Li J., Pang Z. (2022). The elevation gradient of stable isotopes in precipitation in the eastern margin of Tibetan Plateau. Sci. China Earth Sci..

[24] Tsunogai U., Wakita H. (1995). Precursory chemical changes in ground water: Kobe earthquake, Japan. Science.

[25] Wang C.-Y., Manga M. (2010). Hydrologic responses to earthquakes and a general metric. Geofluids.

[26] Skelton A., Liljedahl-Claesson L., Wästeby N., Andrén M., Stockmann G., Sturkell E., Mörth C.-M., Stefansson A., Tollefsen E., Siegmund H. (2019). Hydrochemical Changes Before and After Earthquakes Based on Long- Term Measurements of Multiple Parameters at Two Sites in Northern Iceland—A Review. J. Geophys. Res. Solid Earth.

[27] Han G., Zeng J. (2021). Iron isotope of suspended particulate matter in Zhujiang River, southwest China: Characteristics, sources, and environmental implications. Sci. Total Environ..

[28] Chiodini G., Caliro S., Cardellini C., Frondini F., Inguaggiato S., Matteucci F. (2011). Geochemical evidence for and characterization of CO_2_ rich gas sources in the epicentral area of the Abruzzo 2009 earthquakes. Earth Planet. Sci. Lett..

[29] Favara R., Grassa F., Inguaggiato S., Valenza M. (2001). Hydrogeochemistry and stable isotopes of thermal springs: Earthquake-related chemical changes along Belice Fault (Western Sicily). Appl. Geochem..

[30] Gou X., Yang Y. (2001). Hydrogeochemical evaluation of urban groundwater in Urumqi. Xinjiang. Geology.

[31] Yang Y., Zhang J., Zhang J., Gao Y., Zhou X., Sun X., Wen L., Miao M. (2022). Sedimentary characteristics and main controlling factors of the Middle-Upper Permian and Middle-Upper Triassic in the Bogda Mountain area of Xinjiang, NW China. Pet. Explor. Dev..

[32] Qi R., Song W., Shen R. (2017). Hydrogeological conditions and groundwater quality evaluation of Piedmont gravel plain in Urumqi. Ground Water.

[33] Xiang Y., Sun X., Gao X. (2019). Different coseismic groundwater level changes in two adjacent wells in a fault-intersected aquifer system. J. Hydrol..

[34] Gao X., Li X., Xu Q., Li Y., Zhang X., Cui Y. (2001). Characteristics of earthquake precursory for dissolved CH_4_ in ground water at the No.10 spring, Urumqi. South China J. Seismol..

[35] Chen L., Wang G., Hu F., Wang Y., Liu L. (2014). Groundwater hydrochemistry and isotope geochemistry in the Turpan Basin, northwestern China. J. Arid Land.

[36] Zhong J., Chen S., Wang W., Yan Z., Ellam R.M., Li S. (2020). Unravelling the hydrological effects on patiotemporal variability of water chemistry in mountainous rivers from Southwest China. Hydrol. Process..

[37] Chen S., Zhong J., Li C., Wang W.-F., Xu S., Yan Z.-L., Li S.L. (2020). The chemical weathering characteristics of different lithologic mixed small watersheds in Southwest China. Chin. J. Ecol..

[38] Fournier R.O., Truesdell A.H. (1973). Empirical Na-K-Ca geothermometer for natural waters. Geochim. Cosmochim. Acta.

[39] Jin J., Liu M., Liu Y., Wang J., Gao C., Luo Z., Lu F., Ren Y. (2021). Present-day temperature-pressure field and its controlling factors of the lower composite reservoir in the southern margin of Junggar Basin. Chin. J. Geol..

[40] Ndikubwimana I., Mao X., Zhu D., He Y., Shi Z. (2020). Geothermal evolution of deep parent fluid in Western Guangdong, China: Evidence from water chemistry, stable isotopes and geothermometry. Hydrogeol. J..

[41] Li W., Jiao Y., Zuo Y., Song X., Qiu N. (2014). Effect of deposition rate on geothermal field in Bozhong Depression, Bohai Bay Basin. Chin. J. Geophys..

[42] Craig H. (1961). Isotopic variations in meteoric waters. Science.

[43] Feng F., Li Z., Zhang M., Jin S., Dong Z. (2013). Deuterium and oxygen 18 in precipitation and atmospheric moisture in the upper Urumqi River Basin, eastern Tianshan Mountains. Environ. Earth Sci..

[44] Dobrovolsky I.P., Zubkov S.I., Miachkin V.I. (1979). Estimation of the size of earthquake preparation zones. Pure Appl. Geophys..

[45] Li X., Liu X., Du J., Cui Y., Sun F. (2022). Hydrogeochemical Characteristics of the Haiyuan-Liupanshan Seismic Belt at the Northeastern Edge of the Tibet Plateau. Geofluids.

[46] Taylor H.P. (1977). Water/rock interactions and the origin of H2O in granitic batholiths: Thirtieth William Smith lecture. J. Geol. Soc..

[47] Sun C.J., Li W.H., Chen Y.N., Li X.G., Yang Y.H. (2015). Isotopic and hydrochemical composition of runoff in the Urumqi River, Tianshan Mountains, China. Environ. Earth Sci..

[48] Cai C.F., Mei B., Li W., Zeng F.G. (1997). Water-Rock Interaction in Tarim Basin: Constraints from Oilfieid Water Geochemistry. Chin. J. Geochem..

[49] Garrels R.M., Mackenzie F.T., Gould R.F. (1967). Origin of the chemical compositions of some springs and lakes. Equilibrium Concepts in Natural Water Systems.

[50] Lei X., Wang G. (2022). Is clustered seismicity an indicator of regional stress? Insights from earthquake sequences in Yongning-Luguhu faulted basin, Southwest China. Earthq. Res. Adv..

